# Coding-complete genome sequences of group B equine rotavirus from central Kentucky, USA, reveal circulation of a single genome constellation

**DOI:** 10.1128/mra.00744-25

**Published:** 2025-10-31

**Authors:** Chandika Gamage, Amy Graves, Ganwu Li, Côme J. Thieulent, Udeni B. R. Balasuriya, Jennifer Morrow, Aldana Vissani, Viviana Parreño, Jelle Matthijnssens, Mariano Carossino

**Affiliations:** 1 Department of Pathobiological Sciences, School of Veterinary Medicine, Louisiana State University 70164, Baton Rouge, Louisiana, USA; 2 Louisiana Animal Disease Diagnostic Laboratory, School of Veterinary Medicine, Louisiana State University 70164, Baton Rouge, Louisiana, USA; 3 Equine Diagnostic Solutions, Lexington, Kentucky, USA; 4 Department of Veterinary Diagnostic and Production Animal Medicine, College of Veterinary Medicine, Iowa State University 70724 , Ames, Iowa, USA; 5 Instituto de Virología, CICVyA, Instituto Nacional de Tecnología Agropecuaria (INTA) 42651, Buenos Aires, Argentina; 6 Consejo Nacional de Investigaciones Científicas y Técnicas (CONICET) 62873, Buenos Aires, Argentina; 7 Escuela de Veterinaria, Universidad del Salvador 28213, Buenos Aires, Argentina; 8 Department of Microbiology, Immunology and Transplantation, Laboratory of Viral Metagenomics, KU Leuven, Rega Institute, Leuven, Belgium; DOE Joint Genome Institute, Berkeley, California, USA

**Keywords:** group B rotavirus, equine rotavirus B, bovine rotavirus B, goat rotavirus B, genomic constellation

## Abstract

Equine rotavirus B (ERVB) has caused foal diarrhea in central Kentucky since 2021. Coding-complete genome sequences from 14 strains circulating in 2024 revealed >99% nucleotide identity to the 2021 prototype ERVB strain RVB/Horse-wt/USA/KY1518/2021, with a conserved genomic constellation (G3–P[3]–I3–R3–C3–M3–A4–N3–T3–E3–H3).

## ANNOUNCEMENT

Viruses in the species *Rotavirus betagastroenteritidis,* family *Sedoreoviridae* (formerly group B rotaviruses [RVB]), are non-enveloped with an 11-segment, double-stranded RNA genome encoding six structural proteins (VP1–VP4, VP6, VP7) and at least five non-structural proteins (NSP1–NSP5) (1, 2). Their genome composition is similar to RVA (*R. alphagastroenteritidis*), with most segments predicted to encode proteins with RVA homologs (3, 4). In 2021, an emergent equine rotavirus B (ERVB) was identified in localized outbreaks of foal diarrhea in central Kentucky, USA (5). ERVB has been subsequently detected every foaling season thereafter, and genomic analyses revealed >96% nucleotide identity with bovine and caprine RVB strains, suggesting cross-species transmission (6).

**Fig 1:**
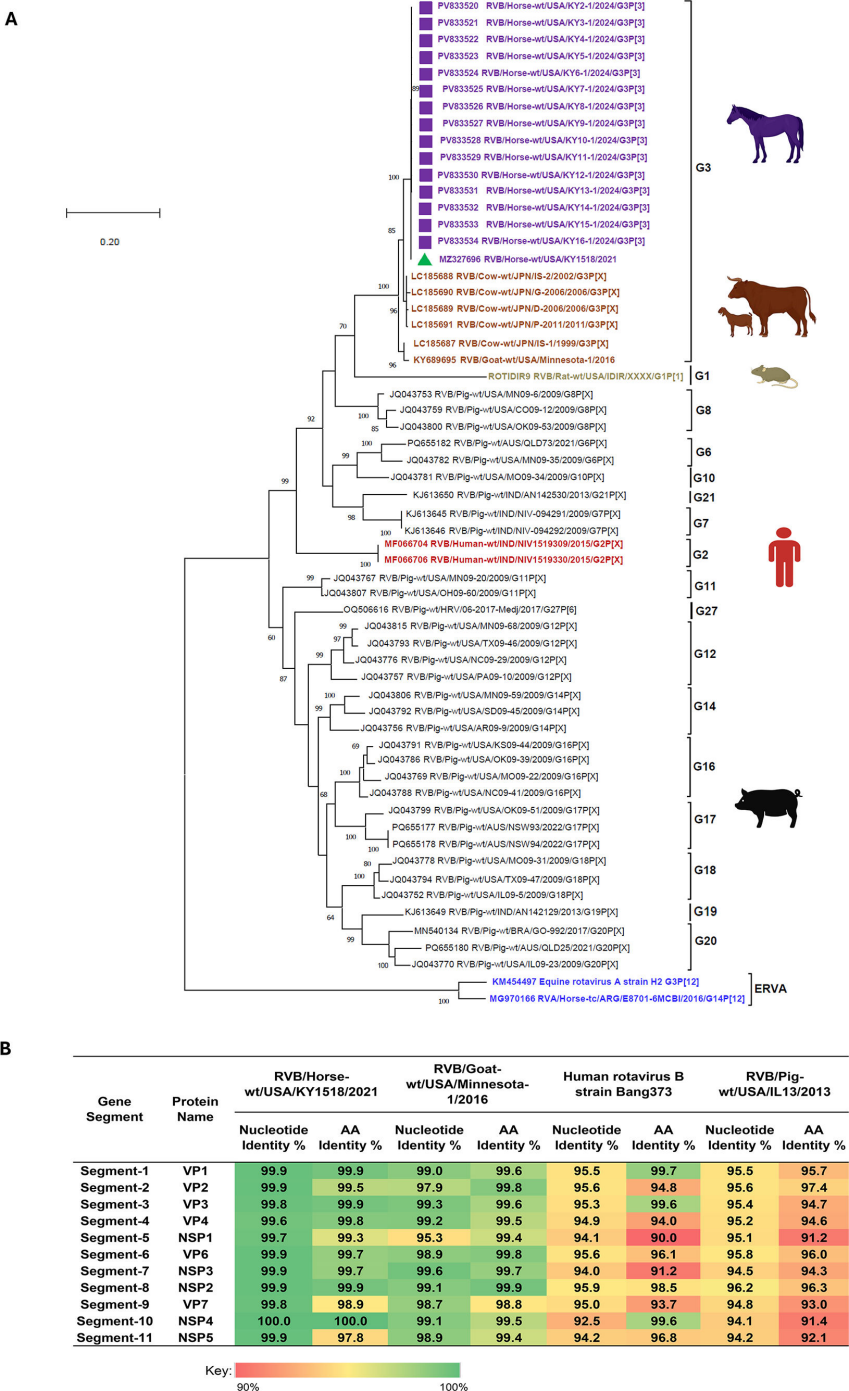
Phylogenetic relationship and comparative sequence analysis of equine rotavirus B strains. (**A**) Representative maximum likelihood phylogenetic tree based on nucleotide sequences from genome segment 9 (outer capsid glycoprotein VP7) of ERVB strains, illustrating the evolutionary relationship with selected RVB reference strains. Fourteen samples yielded coding-complete sequences and were aligned using Geneious Prime 2025.2 (Biomatters Ltd, Auckland, NZ) and phylogenetic trees were constructed in MEGA11 (9) using maximum likelihood with 1,000 bootstraps. Equine rotavirus A was used as an outgroup. Bootstrap values are indicated at major nodes. The scale bar represents the number of nucleotide substitutions per site. Reference sequences were selected from (8), and representative sequences from each established genotype were included to maximize coverage of genotype diversity. (**B**) Nucleotide and amino acid (AA) sequence identity across the 11 genome segments of selected reference rotavirus B (RVB) strains compared to ERVB strains characterized in this study. Nucleotide identity values were derived from multiple sequence alignments of all ERVB genomes with reference strains in Geneious Prime 2025.2. Pairwise distances were calculated for each genome segment, and mean nucleotide and amino acid identities were reported.

During the 2024 foaling season (January–May) in central Kentucky, 971 fecal samples from diarrheic foals were screened for equine equine rotavirus A (ERVA) and ERVB via quadruplex RT-qPCR assay (2), resulting in 96 (9.9%) and 153 (15.8%) positive detections, respectively. Among these, 15 ERVB-positive samples (Ct <22) from different farms were selected for whole-genome sequencing. Total RNA was extracted (MagMAX Pathogen RNA Kit, ThermoFisher Scientific, Waltham, MA), cDNA synthesized (UltraClean ds-cDNA Synthesis Module, Vazyme, Nanjing, China), and DNA libraries prepared (Illumina DNA Prep kit, Illumina, San Diego, CA) and sequenced (Illumina MiSeq i100 Plus platform as 300 bp paired-end reads, with 6.0 × 10⁵ − 1.0 × 10⁶ reads generated at a sequencing depth of 200–500×). Raw reads were pre-processed (Trimmomatic v0.36 (7) and assessed with FastQC (https://www.bioinformatics.babraham.ac.uk/projects/fastqc/). *De novo* assemblies were generated using ABySS (v2.2.4) and SPAdes (v3.13.0); the assembly with higher contiguity and coverage was retained. Where complementary contigs occurred, reads were mapped to the reference strain RVB/Horse-wt/USA/KY1518/2021 (GenBank MZ327688–MZ327698) with BWA-MEM (v0.7.17), and consensus sequences were extracted using SAMtools (v1.7) and curated in IGV (v2.16.0). Each genome assembly had 11 contigs corresponding to each genome segment, with N50 values equal to the segment length. Default parameters were used for all bioinformatic tools. High-fidelity RT-PCR and Sanger sequencing were employed to resolve incomplete coding sequences (https://figshare.com/s/802086006e5a06137504). Coding-complete sequences for all genome segments were obtained from 14/15 ERVB-positive fecal samples (GC content: 34.77%–42.18%). Coding completeness was determined by comparison to the reference strain. Genotype designations were based on established nucleotide identity cut-off and phylogenetic clustering criteria (8). A representative phylogenetic tree (VP7) and nucleotide and amino acid identities are summarized in Fig. 1. ERVB strains showed a conserved genomic constellation (G3–P[3]–I3–R3–C3–M3–A4–N3–T3–E3–H3) (Table 1), consistent with bovine-like RVB strains. Nucleotide identities exceeded 99% compared to the prototype RVB/Horse-wt/USA/KY1518/2021, suggesting limited diversification since 2021 (Fig. 1). The absence of reassortment and very limited antigenic drift underscores the relative genomic stability of ERVB in horses to date. In conclusion, ERVB strains with a single genomic constellation and limited intragenotypic and intergenotypic diversity have circulated among foals in central Kentucky since 2021, with no evidence of reassortment. Continued surveillance is critical to understand ERVB epidemiology, detect emergent variants, assess interspecies transmission, and inform the rational design of ERVB-specific vaccines.

**TABLE 1:** Comparative genome constellations of representative rotavirus B (RVB) strains across different mammalian host species^*c*^

Species	RVB strain	Protein name										
		VP7	VP4	VP6	VP1	VP2	VP3	NSP1	NSP2	NSP3	NSP4	NSP5
Rat	RVB/Rat-wt/USA/IDIR	G1	P (1)	I1	R1	C1	M1	A1	N1	T1	E1	H1
Human	RVB/Human-wt/BG/Bang117	G2	P (2)	I2	R2	C2	M2	A2	N2	T2	E2	H2
Cattle	RVB/Bovine-W/IND/RUBV282/2005	G5	P (3)	I3	R5	C5	NA	A5	N4	NA	NA	H5
	RVB/Bovine-wt/JPN/G-2006/2006/G3PX	G3	P (3)	NA	NA	NA	M3	A3	N3	T3	E3	H3
Pig	RVB/Pig-wt/USA/IL5/2012	G16	P (4)	I13	R4	C4	M4	A8	N10	T4	E4	H7
	RVB/Pig-wt/USA/IL13/2013	G16	P (4)	I13	R4	C4	M4	A8	N10	T4	E4	H7
	RVB/Pig-wt/USA/IL16/2013	G16	P (5)	I13	R4	C4	M4	A8	N10	T4	E4	H7
Goat	RVB/Goat-wt/USA/CA22/2014	G3	P (3)	I3	NA	C3	NA	A4	N3	T3	E3	H3
	RVB/Goat-wt/USA/Minnesota-1/2016	G3	P (3)	I3	R3	C3	M3	A4	N3	T3	E3	H3
Horse	RVB/Horse-wt/USA/KY1518/2021^*a*^	G3	P (3)	I3	R3	C3	M3	A4	N3	T3	E3	H3
	RVB/Horse-wt/USA/KY2-1/2024^*b*^	**G3**	**P (3)**	**I3**	**R3**	**C3**	**M3**	**A4**	**N3**	**T3**	**E3**	**H3**

^*a*^ Reference strain (2021).

^*b*^ Representative 2024 strain from this study.

^*c*^ Shaded cells indicate common genome segments between bovine, goat, and equine RVB. NA: not available. Bolded values highlight a representative ERVB genome constellation derived from this study.

## Data Availability

Primer sequences are available at: https://figshare.com/s/802086006e5a06137504. The raw sequencing data generated and analyzed in this study have been deposited in the National Center for Biotechnology Information (NCBI) Sequence Read Archive (SRA) under the BioProject accession number PRJNA1289110. The raw sequencing reads have been deposited in the Sequence Read Archive (SRA) under the following accession numbers: SRR34444589, SRR34444586, SRR34444583, SRR34444580, SRR34444577, SRR34444591, SRR34444588, SRR34444585, SRR34444582, SRR34444579, SRR34444590, SRR34444587, SRR34444584, SRR34444581, and SRR34444578. This BioProject accession should be referenced in any citations or database searches related to this data set. Coding-complete genome sequences of ERVB from this study have been deposited under the accession numbers PV833400, PV833401, PV833402, PV833403, PV833404, PV833405, PV833406, PV833407, PV833408, PV833409, PV833410, PV833411, PV833412, PV833413, PV833414, PV833415, PV833416, PV833417, PV833418, PV833419, PV833420, PV833421, PV833422, PV833423, PV833424, PV833425, PV833426, PV833427, PV833428, PV833429, PV833430, PV833431, PV833432, PV833433, PV833434, PV833435, PV833436, PV833437, PV833438, PV833439, PV833440, PV833441, PV833442, PV833443, PV833444, PV833445, PV833446, PV833447, PV833448, PV833449, PV833450, PV833451, PV833452, PV833453, PV833454, PV833455, PV833456, PV833457, PV833458, PV833459, PV833460, PV833461, PV833462, PV833463, PV833464, PV833465, PV833466, PV833467, PV833468, PV833469, PV833470, PV833471, PV833472, PV833473, PV833474, PV833475, PV833476, PV833477, PV833478, PV833479, PV833480, PV833481, PV833482, PV833483, PV833484, PV833485, PV833486, PV833487, PV833488, PV833489, PV833490, PV833491, PV833492, PV833493, PV833494, PV833495, PV833496, PV833497, PV833498, PV833499, PV833500, PV833501, PV833502, PV833503, PV833504, PV833505, PV833506, PV833507, PV833508, PV833509, PV833510, PV833511, PV833512, PV833513, PV833514, PV833515, PV833516, PV833517, PV833518, PV833519, PV833520, PV833521, PV833522, PV833523, PV833524, PV833525, PV833526, PV833527, PV833528, PV833529, PV833530, PV833531, PV833532, PV833533, PV833534, PV833535, PV833536, PV833537, PV833538, PV833539, PV833540, PV833541, PV833542, PV833543, PV833544, PV833545, PV833546, PV833547, PV833548, PV833549, PV833550, PV833551, PV833552, PV833553, PV833554, PV833555, PV833556, PV833557, PV833558, PV833559, PV833560, PV833561, PV833562, PV833563, PV833564.
